# The Pictorial–Semantic–Task Framework for Understanding Graph Comprehension

**DOI:** 10.3390/jintelligence14020028

**Published:** 2026-02-12

**Authors:** Evelyn Hsin-I Tsai, Yoojin Hahn, Robert S. Siegler

**Affiliations:** Department of Human Development, Teachers College, Columbia University, New York, NY 10027, USA; yh2729@tc.columbia.edu

**Keywords:** graphs, graph comprehension, math learning, data visualization

## Abstract

Graphs are used in school, many occupations, and daily life, yet many people struggle to interpret them accurately. To help identify sources of difficulty in graph comprehension, we propose the Pictorial–Semantic–Task Framework. In it, we argue that accurate interpretation of graphs requires integrating pictorial variables (e.g., slope direction, graph format, data points) with semantic variables (e.g., titles, labels, scales, variable types) to determine what the graph represents. Many errors arise because readers fail to coordinate these two sources of information, often basing interpretations solely on pictorial variables. The present theoretical synthesis presents the basic analysis underlying the Pictorial–Semantic–Task Framework and an integrative review of relevant findings from graph encoding, extrapolation, and comparison tasks. The findings show that people encode and recall pictorial information far more accurately than semantic information, and often base interpretations solely on visual patterns even when semantic features call for a different conclusion. Analyses of U.S. textbooks and mass media reveal potential sources of these biased interpretations: systematic imbalances in the types of semantic information provided in textbooks and media seem likely to contribute to biases, emphasizing visual over semantic cues. By describing how perceptual and conceptual processes interact during graph comprehension, we aim to advance theories of cognitive processing in the context of graph comprehension and to derive educational implications for helping children interpret graphs more accurately.

## 1. The Pictorial–Semantic–Task Framework for Understanding Graph Comprehension

Understanding graphs is essential in school, daily life, and the workplace. Children often encounter graphs in science and mathematics classes in elementary, middle, and high school (Donnelly-Hermosillo et al., 2020; Planinic et al., 2012). Adults often encounter graphs in everyday life, for example, to understand trends in their finances; to track the spread of viruses and diseases; and to understand social issues such as changes in drug use, teenage pregnancies, and crime (Franconeri et al., 2021). Reflecting the ubiquity and importance of graphs, the Common Core State Standards (CCSS) emphasizes the importance of graph literacy, as does the Next Generation Science Standards (NGSS) and both U.S. (NAEP) and international (PISA and TIMSS) educational assessments (Common Core State Standards Initiative, 2010; NGSS Lead States, 2013; OECD, 2023).

Despite the widespread use of graphs and recognition of their importance, many children and adults have difficulty interpreting and using them, for example, to calculate slopes, extrapolate values, and compare trends (Binali et al., 2022; Ciccione & Dehaene, 2021; Lai et al., 2016). For instance, only 29% of U.S. eighth graders correctly calculated the mean of the points in a line graph on a standardized international test of mathematical understanding (TIMSS, 2013). Similarly, only 44% of eighth graders correctly described the differences between the information presented in two graphs in Ozmen et al. (2020). Misconceptions about slopes, as well as difficulties linking graph features to real-world concepts, persist into college (Binali et al., 2022; Gardner et al., 2024). These findings are concerning, given the growing need to interpret graphs both in and out of school (Börner et al., 2019; Lammers et al., 2020). Yet the mechanisms underlying failures in graph comprehension are not well understood.

In this article, we present the Pictorial–Semantic–Task Framework, which provides a theoretical analysis of graph comprehension. We propose that elements of graphs can be grouped into two categories: pictorial variables, which reflect graphs’ visual characteristics, and semantic variables, which convey graphs’ content. Although prior research has often examined how viewers process pictorial variables, far less attention has been devoted to how people process semantic information and how they integrate pictorial and semantic information. The Pictorial–Semantic–Task Framework also recognizes the importance of adopting strategies that meet task requirements for successful use of graphs and that both children and adults often fail to adopt such strategies. Below, we describe the theoretical framework, then examine research relevant to it, and then note some of its educational implications.

## 2. The Pictorial–Semantic–Task Framework

Graph comprehension has been characterized as a hierarchy of interpretive levels (reading the graph, identifying relations among its elements, and drawing inferences from data (Curcio, 1987)); as a set of perceptual processes that activate graph schemas (Pinker, 1990); and as an interaction between visual processing and prior knowledge (Shah & Hoeffner, 2002). However, none of these approaches specify which types of graph information people extract and encode most accurately, how different features are weighed during comprehension, and why incorrect interpretations are so common. The Pictorial–Semantic–Task Framework is an attempt to address these issues.

**Figure 1:**
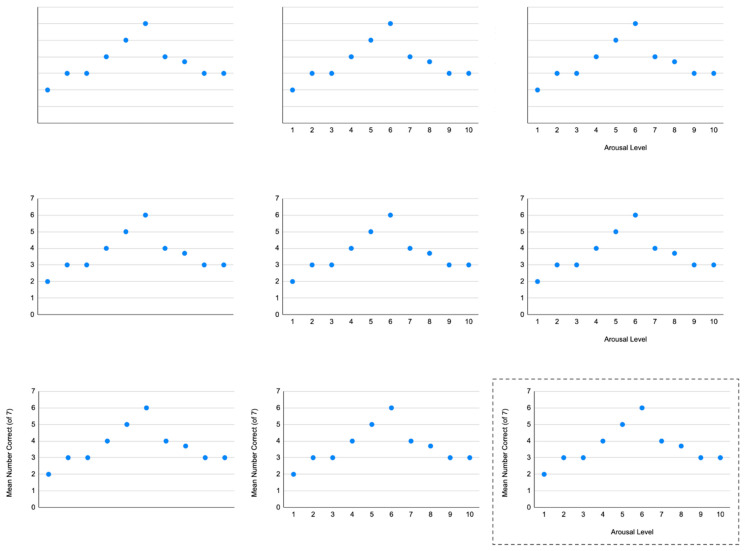
Effect of Semantic and Pictorial Variables on Interpretability of Graphs.

The necessity of processing semantic, as well as pictorial, variables is illustrated in the matrix in Figure 1. The graph at the top left shows that values of the y-axis variable rise and then fall with changes in values of the x-axis variable. However, without semantic context, the graph is meaningless; there is no way to know what the pattern represents.

The center panel in the matrix adds numeric scales to the x-axis (1 to 10) and y-axis (0 to 7). This tells readers a little about the graph’s possible meaning, but only a little.

In contrast, in the bottom-right panel, where both axes have labels and scales, the graph can be interpreted both qualitatively and quantitatively. This panel shows that with increases in arousal, mean accuracy on seven problems initially increased from 2 to 6, but with further increases in arousal, mean accuracy decreased from 6 to 3 items. Interpreting the scale of arousal and the difficulty of performance would require reading the text, but for readers who have done so, the graph in the bottom-right panel provides an easy-to-understand summary of the findings.

As this example illustrates, two graphs with identical visual appearances can convey entirely different meanings, depending on their semantic content. Think of the difference if the y-axis label were “Mean Incorrect” instead of “Mean Correct.” Thus, graph comprehension requires more than visual decoding of pictorial features; it also depends on understanding the semantic content conveyed by the axes, title, caption, etc.

Different tasks elicit different graph processing strategies. For instance, tasks that ask students to identify slope direction promote attention to pictorial trends, whereas tasks that ask students to explain the graph’s main message require joint processing of pictorial and semantic information. Thus, the specific task mediates the role of pictorial and semantic features in graph processing. Successful comprehension depends not only on encoding visual and semantic information but also on identifying the information relevant to the task. Because tasks vary in cognitive demands, graph comprehension reflects dynamic interactions among pictorial, semantic, and task variables.

A central prediction of the Pictorial–Semantic–Task Framework is that both children and adults rely more on pictorial cues and less on semantic and task cues than is optimal for graph comprehension. It is not the case that they cannot rely on semantic and task cues—if those are strong enough, people rely on them. However, biases in processing, based at least in part by biases in the graphs that people encounter during school and adulthood, lead to reliance on pictorial cues in many situations where that is inappropriate.

In the sections that follow, we first review previous work on how pictorial, semantic, and task variables influence graph comprehension and on how graph interpretation errors often stem from an overreliance on pictorial cues and insufficient attention to semantic content. Next, we examine new work from our lab that tests hypotheses based on the Pictorial–Semantic–Task Framework and integrate the prior and new findings in the context of the framework. We conclude by discussing implications of the framework and findings for education and directions for future research.

## 3. Effects of Pictorial, Semantic, and Task Variables on Graph Comprehension

Comprehending graphs requires synthesizing information from pictorial and semantic features. Pictorial variables refer to such visual characteristics as trend direction, strength and shape of the relation (e.g., linear, curvilinear), format (e.g., bars, lines, dots, pies), and number of data points. These features collectively create each graph’s visual structure. Semantic variables convey the content being graphed through titles, captions, x- and y-axis labels, numerical and categorical scales, units of measurement, and variable types (e.g., continuous, categorical). Together, semantic and pictorial features create a graph’s meaning.

Task variables indicate which pictorial and semantic features of graphs are relevant in the immediate situation. Misunderstanding the problem specified by the task often leads to incorrect answers by focusing attention and memory on features of graphs that are irrelevant or misleading. Identifying the information needed to answer specific questions imposes another level of difficulty on the graph comprehension process.

Next, we examine effects of pictorial, semantic, and task variables on graph comprehension.

### 3.1. Effects of Pictorial Variables

Gestalt principles describe several influences on graph comprehension. In line graphs, the principles of continuity and connectedness suggest that lines are perceived as cohesive and uninterrupted, supporting the tracking of changes in the dependent variable over time (Wertheimer & Riezler, 1944; Strobel et al., 2016; J. Zacks & Tversky, 1999). In bar graphs, the principle of proximity suggests that bars positioned close together are perceived as a group, whereas bars spaced farther apart are perceived as separate, aiding viewers in mapping the bars to their corresponding categories (Shah et al., 1999). Shared color and shape are additional Gestalt variables that can facilitate comprehension by promoting attention to graph elements with common visual features (Peebles & Ali, 2015; J. M. Zacks & Franconeri, 2020).

Although pictorial features can support rapid perceptual judgments, they can also mislead viewers when they conflict with semantic features. For example, a steep line ascending from left to right may be read as connoting rapid growth, but if the y-axis is labeled “Errors,” the slope reflects a steep decline in performance. Moreover, distortions in pictorial variables, including extraneous depth cues, task-irrelevant data, exaggerated bar width, and disproportionate scaling making small effects look large, are common in graphs appearing in media and can hinder accurate interpretation (Doan, 2021; Fischer, 2000; Kwon et al., 2021; Tufte, 1983). Indeed, hindering accurate interpretation and promoting preferred ones may often be the purpose of such graphs. For example, truncating the y-axis to display a narrow range of values can make small numerical differences appear substantial, leading viewers to erroneously infer dramatic change when differences are minimal (Correll et al., 2020; Pandey et al., 2015; Yang et al., 2021).

### 3.2. Effects of Semantic Variables

Semantic features shape viewers’ perception of a graph’s main message. Consider effects of titles and captions. Kong et al. (2019) found that participants were more likely to recall the message conveyed by a graph’s title than the plotted data, even when the data contradicted the title. Wanzer et al. (2021) reported that longer, more descriptive titles did not improve graph comprehension accuracy relative to succinct titles, indicating that elaboration does not necessarily enhance understanding. In a third example, Kim et al. (2021) discovered that when captions emphasized visually salient features (e.g., a major trend), participants remembered the caption as the graph’s primary message. However, when captions emphasized less salient details (e.g., minor fluctuations in trends), participants largely disregarded the caption and instead recalled visually prominent patterns as the key takeaway.

Axis labels provide essential semantic information by specifying the variables, units, and relations being depicted. Clear, concise labels positioned near the relevant data support faster and more accurate interpretation than labels placed farther away (Pinker, 1990). However, during the COVID-19 pandemic (and at other times as well), many graphs in media omitted axis labels or used inconsistent axis scaling, producing distorted impressions of trends and their strength (Doan, 2021; Engledowl & Weiland, 2021; Kwon et al., 2021).

Combinations of semantic and pictorial variables often determine which graph format (bars, lines, pies, etc.) most effectively conveys data. Bar graphs, for instance, are well suited for categorical variables (e.g., ones showing values for different countries, medications, or companies), because spatially separated bars signal discrete groups and are interpreted more accurately when the x-axis represents categorical rather than continuous values (J. Zacks & Tversky, 1999). In contrast, line graphs are more appropriate for continuous x-axis variables (e.g., time or dosage), as lines imply continuity (Postigo & Pozo, 2004; J. Zacks & Tversky, 1999). On the other hand, using line graphs to represent nominal categories can be misleading, as connecting lines may falsely imply ordered relations among discrete groups. Thus, aligning semantic and pictorial variables is important for effective communication of data.

Studies using eye tracking methods to examine processing of graphs (e.g., Boels et al., 2024; Lauer & Sanchez, 2023) have shown that greater attention to relevant semantic information, such as variable names and numerical values on the x- and y-axes, is consistently associated with more accurate comprehension (Vila & Gomez, 2016; Keller & Junghans, 2017). Compared with novices, skilled graph readers fixate less on perceptually salient visual features and more on semantic features (e.g., axis scales) and show more integrative eye-movement patterns across axes, legends, and data (Atkins & McNeal, 2018; Harsh et al., 2019; Ruf et al., 2023). These findings from studies using eye tracking again demonstrate the importance of integrating pictorial and semantic information for accurate graph comprehension.

### 3.3. Effects of Task Variables

Within the Pictorial–Semantic–Task Framework, task variables act as critical moderators: they define goals and specify features that are essential to accurate comprehension. Different tasks create distinct processing demands, requiring viewers to encode some information and allowing them to ignore other information (Friel et al., 2001; Shah & Hoeffner, 2002). For instance, if a graph presents changes in income over time in different states, and the task is to identify which state had the greatest relative increase in that time, viewers must integrate pictorial and semantic information to calculate proportional change. People who focus only on a pictorial feature (e.g., height of the highest bar) or a single semantic value (e.g., largest income) will usually err.

Performance also varies with how compatible the graph format is with the task. Converging findings from behavioral, self-report, and eye-tracking studies show that point comparison tasks are most accurate with bar graphs, because discrete bars facilitate identification of precise values (Pinker, 1990). In contrast, tasks requiring identification of trends are solved more quickly and accurately with line graphs, as the steepness of the line facilitates identification of the rate of change (Shah & Freedman, 2009; Strobel et al., 2016; J. Zacks & Tversky, 1999). In a third case, proportion judgment tasks are facilitated by pie charts, whose circular structures highlight part–whole relations (Hollands & Spence, 1992).

Recent studies in our lab on extrapolation from graphs, encoding of graph content, and exposure to different types of graphs have extended these prior findings and tested predictions of the Pictorial–Semantic–Task Framework. We next present this new research.

## 4. Extrapolating from Graphs

Extrapolating from graphs requires predicting unspecified values from data displays. Extrapolations tend to be more accurate for linear than nonlinear graphs, partly because people often misinterpret nonlinear patterns as linear, which leads them to underestimate growth that is more than linear and overestimate growth that is less than linear (Confrey & Smith, 1995; Van Dooren et al., 2004). Extrapolation also tends to be more accurate when graphs display stronger relations between variables, include more data points, have ascending slopes, or use a point graph format, demonstrating that pictorial features shape interpretation even when semantic information remains constant (Ciccione & Dehaene, 2021; Reimann et al., 2020; Reimers & Harvey, 2024).

Most studies have examined extrapolation with continuous x-axis variables such as time, speed, price, or dosage; little is known about how people extrapolate from graphs with categorical x-axis variables, such as city, region, company, or medication. Hahn and Siegler (2025) proposed that viewers often extrapolate based solely on pictorial trends, ignoring semantic information about the variable type. This strategy is appropriate when the x-axis variable is continuous, but inappropriate when it is categorical. For example, in a bar graph of class members organized from the shortest to the tallest child, relying entirely on pictorial cues suggests that the next child whose height is graphed will be the tallest, continuing the upward trend. This inference is invalid because categorical variables have no inherent order; in such cases, the most valid extrapolation is the mean of the previous values (Theocharis et al., 2019).

Hahn and Siegler (2025) assessed adults’ extrapolation accuracy while manipulating both semantic features (categorical vs. continuous x-axis variable) and pictorial features (relation strength, slope direction, number of points, and graph format). Consistent with the prediction of the Pictorial–Semantic–Task framework, participants’ extrapolations relied more on pictorial than semantic variables, resulting in accurate extrapolations when the x-axis represented continuous variables and inaccurate extrapolations when the x-axis represented categorical variables.

A second study by Hahn and Siegler examined effects of a task variable on extrapolation from graphs. Graduate students completed the same extrapolation task as in the first study but were instructed to base extrapolations on the trend of the data, the mean of the data, or received no instructions. Results from the no-instruction condition replicated the findings from the first study. Explicit instruction to extrapolate using the mean substantially improved accuracy for graphs with categorical x-variables, suggesting that the inaccurate extrapolations with categorical x-axis variables in the prior experiment stemmed from a misapplied pictorial default strategy, not an inability to identify the mean. On the other hand, instructions to extrapolate from the mean produced less accurate extrapolations on graphs with continuous variables. Pictorial cues that emphasized the trend (steeper slopes, more data points, line formats) amplified errors in the no-instruction condition when the x-variable was categorical, but improved accuracy when the x-variable was continuous.

These findings supported two central claims of the Pictorial–Semantic–Task Framework: accurate graph comprehension requires integrating pictorial and semantic information, and task variables can influence relative emphasis on pictorial and semantic variables. When pictorial trends align with semantic variables, they yield accurate inferences. When the two types of cues conflict, however, overreliance on pictorial cues can mislead even highly educated viewers.

## 5. Encoding of Pictorial and Semantic Variables in Graphs

Encoding graph content is necessary but not sufficient for effective use of the encoded content. That is, the information viewers attend to and register is needed to make accurate judgments and draw correct conclusions, but encoding the content does not necessarily lead to such outcomes.

To investigate what information people encode, retain, and use when interpreting graphs, Tsai et al. (2025) conducted four cross-cultural, cross-age studies with U.S. and Chinese 12-year-olds and adults. Participants viewed a graph for 15 s and then judged whether a second graph conveyed the same or different information. Across trials, graphs differed either in semantic features (e.g., axis labels or scales) or pictorial features (e.g., trend direction or steepness). To differentiate failures of encoding from failures of retrieval, participants completed the task under both a viewing condition (the two graphs shown side-by-side) and a retrieval condition (the first graph was deleted before comparison to a second graph).

Across both ages and cultures, a consistent pattern emerged: pictorial features were encoded and retrieved far more accurately than semantic features. Accuracy for encoding and retrieving pictorial features exceeded 85% in every group. In contrast, accuracy in detecting semantic differences in the retrieval condition was only 36% for U.S. children and 54–61% for U.S. adults and Chinese adults and children. When pictorial features of two graphs were the same, people frequently judged the graphs as identical even when axis labels or scales differed.

The superior performance with simultaneous rather than successive displays of the graphs seemed likely to reflect limits in visual working-memory storage capacity. Such limits would be expected to have little effect when the graphs were simultaneously present, but to have large effects when the graphs were presented successively. On the other hand, the large differences between Chinese and U.S. children’s performance in the retrieval condition suggested that when analyzing graphs, the Chinese children allocated cognitive resources more flexibly and strategically than U.S. children. Thus, differences in adjusting to task requirements, as well as working memory limits, seemed likely to contribute to the effects, as has been found in other domains as well (Fougnie et al., 2016; Ouwehand et al., 2025; Ye et al., 2019).

As predicted by Fuzzy Trace Theory (Reyna & Brainerd, 1995), the patterns of encoding and retrieval accuracy, especially among U.S. children but in other groups as well, emphasized gist over verbatim information. In the graph comprehension context, gist representations, such as those of overall trend direction and graph shape, were processed more accurately than verbatim information, such as scale values and units. This again was consistent with findings in a wide range of domains (Reyna & Brainerd, 2011).

Tsai et al. (2025) examined what information people cited on a task where they were explicitly asked to verbally describe the information in graphs rather than classify two graphs as identical or different. Participants viewed a graph for 20 s and then described it either with the graph present or from memory. Because the task of describing requires attention to the graph’s content (Tsuji & Lindgaard, 2014; Xi, 2010), semantic information was expected to dominate the verbal descriptions, unlike on the same/different judgment task used in the previous experiment.

This expectation was borne out, but only partially. Participants in all groups accurately described some pictorial features (trend direction) and qualitative semantic features (x- and y-axis labels), likely because these features convey the graph’s gist. However, quantitative semantic features (x- and y-axis scales) were reported less accurately. Moreover, recall of quantitative semantic information was far lower in the memory than in the viewing condition, while accuracy for pictorial and qualitative semantic features remained stable. Thus, both qualitative semantic information and pictorial information were recalled quite well when the task involved explicit verbal comparisons of graphs, but quantitative semantic information was not recalled well even in this context.

Cultural and developmental differences also emerged. Chinese children’s performance approached that of Chinese adults, whereas U.S. children showed substantially lower accuracy than U.S. adults. For instance, Chinese adults and children described qualitative semantic features with similarly high accuracy (93% vs. 89%), whereas U.S. adults and children showed a much larger gap in accuracy in describing those features (93% vs. 67% correct). For quantitative semantic features, accuracy among Chinese adults and children was again similar (62% vs. 65%), whereas accuracy of U.S. children again lagged far behind that of U.S. adults (46% vs. 77% correct).

The developmental differences observed in the U.S. sample are consistent with Cognitive Load Theory (Ouwehand et al., 2025). Encoding and interpreting semantic graph features, especially quantitative features such as scale values and units, requires maintaining multiple symbolic elements in working memory and integrating them with pictorial patterns, creating high intrinsic cognitive load. The difficulty of maintaining all of this information is likely greater for children whose working-memory capacity is still developing, than for adults. On the other hand, the excellent performance of Chinese children demonstrates that the difficulties imposed by the relatively high cognitive load of the graph recall condition can be overcome with high quality math instruction and a culture that emphasizes mathematical proficiency.

Together, these findings suggest that perceptual encoding and verbal description of graph content may involve partially distinct cognitive processes. Across tasks, quantitative semantic information is consistently the hardest to encode and remember for children and adults in both China and the U.S. Even when participants could describe qualitative semantic features verbally when asked to do so, they did not always use those features when judging whether two graphs were equivalent Thus, differences among tasks shapes what information is encoded, processed, and applied on graph comprehension problems.

## 6. Exposure to Different Types of Graphs and Graphing Tasks Influences Processing of Semantic and Pictorial Variables

How do people come to process graphs as they do? Research in other areas of mathematics suggests that input from textbooks and media helps shape the processing (Braithwaite et al., 2017; Horsley & Sikorová, 2014).

### 6.1. Relations of Textbook Graphs to Children’s Graph Comprehension

To examine potential effects of textbook problems on children’s graph comprehension, Tsai and Siegler (2025) identified all illustrations in the 5th to 8th grade volumes of three widely adopted U.S. math textbooks that met our definition of graphs. Graphs were defined as illustrations that pictorially represent numerical relations between two or more variables and include labels on both the x- and y-axes. A total of 1206 graphs met these criteria. Photos, tables, number lines, and grids displaying areas and perimeters were excluded from the analysis. A formal coding scheme was used to classify the types of x- and y-axis variables, graph format, slope magnitude, number of data points, and task type. Interrater reliability between the first author and a research assistant coding a sample of 100 graphs from textbooks was high (κ = 0.93).

Across grades, textbooks contained far more problems asking students to complete basic tasks, such as identifying individual data points, than more advanced tasks, such as determining the means of data points and calculating slopes. In fact, more than half of all graph problems in the three 5th to 8th grade textbooks could be solved through point identification alone. These findings reveal that U.S. middle school textbooks offer limited practice with even moderately advanced graph skills, including those commonly required on standardized mathematics assessments of 8th grade math achievement, such as calculating rates and proportions.

In a second study (Tsai & Siegler, 2025), middle-school students were presented the five main types of graph problems observed in the math textbooks: identifying points, determining trends, calculating slopes, comparing graphs, and plotting graphs from point coordinates. Children’s performance paralleled the relative frequency of graph problems in textbooks in several ways. Students were more accurate on tasks that appeared more frequently in textbooks—they located points correctly on 89% of trials—than on problems that were less common, such as determining trends (75%) and calculating slopes (70%). The middle school students were also more accurate on problems involving positive slopes than negative ones (80% vs. 74%), consistent with textbooks presenting ascending trends much more often than descending ones, even though the interpretative requirements seem the same. Further, the students were more accurate at identifying points in bar graphs than line graphs, and more accurate at identifying slopes in line graphs than bar graphs, again perhaps because these combinations of formats and tasks are more common in textbooks than other pairings.

A similar pattern appeared in graph comparison tasks that were examined in the same study. In the math textbooks, nearly all side-by-side graph comparison problems involved changes in pictorial features (i.e., trend direction, steepness, or shape). Fewer than 10% involved changes in semantic features (i.e., x- and y-axis labels and scales) (Tsai et al., 2025). This imbalance may wrongly signal to students that semantic information, such as variables and scales, is fixed and therefore irrelevant to comparing and interpreting graphs. Consistent with this possibility, when U.S. children were tested on graph comparison tasks, they were highly accurate at detecting pictorial differences between graphs, but they frequently overlooked differences in semantic variables (Tsai et al., 2025). The implication is that students may pay relatively little attention to semantic features, in part because textbooks emphasize pictorial variation while rarely modeling semantic variation.

These relations between frequency of different types of graph problems in U.S. math textbooks and graph comprehension align with findings from whole number, fraction, and decimal arithmetic. In all of those areas, less frequent problem presentation in textbooks predicts weaker performance on the less frequently presented problems. In arithmetic, causal as well as correlational connections have been established (Braithwaite & Siegler, 2023; McNeil et al., 2015). Whether such causal relations are also present between textbook input and graph comprehension remains to be established, however.

### 6.2. Relations of Graphs in Newspapers to Adults’ Graph Comprehension

To broaden analyses beyond textbooks to materials that adults encounter, Hahn and Siegler (2025) examined graphs published in four major U.S. news outlets (New York Times, Washington Post, Wall Street Journal, and USA Today) in a week of 2023. Media graphs exhibited distributions similar to those in textbooks: continuous x-axis variables were far more common than categorical x-axis variables, and compared with graphs that had categorical x-axis variables, those with continuous variables were more likely to have line formats, ascending slopes, and larger numbers of data points.

Such input from media, as well as prior input from textbooks, may contribute to the relative accuracy of adults’ extrapolations on different types of graphs. Adults in Hahn and Siegler (2025) extrapolated more accurately from graphs with continuous x-axis variables, the type more commonly presented in both textbooks and news media, than from categorical graphs, which appeared less often. Likewise, the predominance of ascending trends in both types of input may contribute to adults’ greater accuracy extrapolating from ascending than descending graphs. Errors extrapolating from graphs with categorical variables appear to stem from reliance on pictorial trends—a strategy reinforced by textbooks and media presenting graphs with continuous x-axis variables far more often than graphs with categorical variables.

Analyses of side-by-side media graphs also revealed imbalances similar to those in textbooks: Almost all comparisons involved differences in pictorial variables, whereas semantic differences were rare. This distribution may reinforce attention to pictorial features and inattention to semantic ones.

Textbook and media graphs differed somewhat in how they present semantic information. Media graphs nearly always included titles, which typically served as the main source of semantic context, whereas only 53% of textbook graphs included titles. On the other hand, textbooks more consistently labeled axes (94% of x-axes and 82% of y-axes), whereas media graphs often omitted axis labels (only 22% of x-axes and 46% of y-axes were labeled).

## 7. Educational Implications of the Pictorial–Semantic–Task Framework

Despite the increasing prevalence of graphs in education, popular media, and professional journals, many people struggle to interpret graphs accurately. Guided by the Pictorial–Semantic–Task Framework, we propose three avenues for improving graph literacy: (1) explicit instruction in implications of semantic features for interpreting graphs, (2) increased exposure to graph types that are relatively rare in textbooks and newspapers, and (3) implementation of interventions aimed at improving aspects of graph comprehension that many children and adults do not understand.

### 7.1. Emphasize the Importance of Semantic Features

Instruction in graph comprehension tends to emphasize pictorial information, as illustrated in the high prevalence of point identification problems and questions such as whether a line slopes upward or downward. However, encoding semantic information is equally important. Instruction should explicitly teach students how to locate, interpret, and integrate semantic and pictorial information, and how to draw inferences from the combined information in graphs. Activities could include classifying variable types (categorical vs. continuous), interpreting the effects of varying units on the appearance of graphs, connecting semantic features to visual elements, and noting cases in which pictorial trends often lead to incorrect interpretations.

For example, introducing conflicts between pictorial and semantic cues—such as pairing a steep upward line with axis labels that contradict the visual impression—could help students learn to integrate the cues. Similarly, comparing graphs with identical visual patterns but differing semantic information can help students understand the importance of encoding and applying semantic information during graph comprehension.

### 7.2. Increased Exposure to Underrepresented Graph Types

Greater exposure to types of graphs that are rarely shown in textbooks—for example, graphs with categorical variables and nonlinear trends—could provide opportunities for students to learn how to interpret these types of graphs. Many problems with U.S. children’s mathematics learning stem from intractable features of society, such as poverty and minimal cultural emphasis on the importance of most children learning math. Textbooks, by contrast, could be changed relatively easily, for example, by presenting types of problems that children find difficult more often.

Even if textbooks remain as they are, teachers could enrich instruction by incorporating a greater number of more advanced problems, such as those involving slope calculation and best-fitting lines. Emphasizing the concepts underlying solution procedures, as well as problem-solving procedures, would likely heighten the effects of such instruction.

### 7.3. Graph Comprehension Interventions

Despite many children and adults having weak graph comprehension skills, few empirically validated graph comprehension interventions have been performed. However, an intervention using Smart Graphs—a platform that provides contextualized tasks, guided visual cues, and scaffolded feedback—illustrates the potential of interventions to improve graph comprehension. The intervention yielded a 49% pre–post improvement in percent correct interpretations of graphs in a randomized controlled trial with eighth- and ninth-grade students (Zucker et al., 2014). It is unclear why subsequent studies have not built on these impressive results; more studies of effective interventions are clearly needed.

## 8. Limitations and Future Directions

Although the Pictorial–Semantic–Task Framework offers a useful lens for understanding graph comprehension, several limitations should be noted. First, the empirical evidence reviewed here primarily focused on line and bar graphs with categorical or continuous variables. Other semantic variations, such as whether the x-axis represents familiar or unfamiliar variables, and the impact on comprehension of conveying semantic information through titles, captions, axis labels, scale values, or some combination of these, are also in need of study.

Second, research conducted within the Pictorial–Semantic–Task Framework has not examined how individual and national differences, such as those in spatial reasoning, math anxiety, and metacognition, influence graph comprehension. Future research should examine whether these characteristics affect how people attend to and integrate semantic and pictorial information and adjust to task variations.

Third, existing analyses linking textbook and media input to graph comprehension have been correlational. Experimental studies in which people are randomly assigned to receive different distributions of graph problems are needed to test whether relations between the frequency of such problems and graph comprehension are causal as well as correlational.

## 9. Conclusions

The Pictorial–Semantic–Task Framework conceptualizes graph comprehension as a process requiring coordinated processing of pictorial and semantic features to adapt to the goals of specific tasks. To comprehend a graph, viewers often must go beyond pictorial variables and process titles, captions, axis labels, axis scales, and other semantic variables that help define the meaning of the graph. This framework helps explain why certain errors persist despite years of schooling: People often rely exclusively on pictorial patterns in cases that also require use of semantic information, leading to erroneous interpretations of the graphed data. Adapting to task requirements requires strategies that focus on all information relevant to the task, semantic as well as pictorial and quantitative semantic as well as qualitative semantic information. Viewing graph comprehension within the Pictorial–Semantic–Task framework not only provides a framework for analyzing graph comprehension but also suggests hypotheses about where processing of graphs tends to go wrong, how graphs in textbooks and media may contribute to those tendencies, and how instruction in graph comprehension might be improved.

## Data Availability

No new data were created or analyzed in this study. Data sharing is not applicable to this article.
